# The price of care: alcohol misuse as a moderator of financial hardship and mental health outcomes of U.S. women caregivers

**DOI:** 10.1007/s00737-025-01672-0

**Published:** 2026-01-28

**Authors:** Heather DeGrande, Luis Enrique Espinoza

**Affiliations:** Texas A&M University-Corpus Christi, Corpus Christi, TX 78412 United States

**Keywords:** Women caregivers, Financial hardship, Alcohol misuse, Mental health

## Abstract

**Purpose:**

Financial hardship and alcohol misuse are well-established predictors of caregiver mental health. The purpose of this study was to examine if alcohol misuse influenced the associations between financial hardship and mental health outcomes (depression diagnosis and poor mental health days).

**Methods:**

A retrospective data analysis was conducted from 4,212 U.S. women caregivers utilizing 2023 Behavioral Risk Factor Surveillance System dataset.

**Results:**

Alcohol misuse when measured dichotomously was not independently associated with depression diagnosis or poor mental health days. However, more frequent alcohol misuse substantially strengthened the food insecurity–psychological distress relationship among women caregivers. Financial hardship—particularly food insecurity—was strongly associated with depression and more days in the past month with worse mental health, especially among women with lower income, lower educational attainment, and heavier caregiving responsibilities.

**Conclusions:**

This study contributes to international research by demonstrating the value of using frequency-based rather than binary substance use measures, thereby enhancing data comparability across health systems. Findings align with evidence from countries that economic vulnerability and maladaptive coping, such as alcohol misuse, can have negative impacts on caregiver well-being. Trauma-informed, harm-reduction, and culturally sensitive public health strategies could reduce both financial and behavioral risks for substance misuse and mental health symptoms among women caregivers globally.

## Article Highlights

- Women caregivers under financial hardship experience worse mental health symptoms.
- Food insecurity is a major stress for these caregivers.
- Alcohol misuse frequency, rather than binary misuse intensified poor mental health among food insecure women caregivers.
- Findings highlight the need for better financial and mental health support for women caregivers.

Caregivers face multifaceted challenges, which can affect caregivers’ physical, emotional, and financial well-being (Krämer and Bleidorn 2024; Lalani et al. 2025; National Alliance for Caregiving [NAC] & The American Association of Retired Persons [AARP], 2020). Challenges related to caregiving tend to be distributed unequally, as women provide the majority of unpaid care globally (Basu and Berkowitz 2022). In particular, women perform the majority of unpaid care work worldwide (76.2%) (International Labour Organization [ILO], 2018). Caregiving typically is completed by an adult who provides unpaid support to a family member, friend or other person living with a disability, chronic illness or age-related need and can include household chores, personal care and/or care coordination (NAC & AARP, (National 2020); Schulz and Eden 2016).

Caregiving can often become coupled with monetary distress, which has been shown to be associated with high rates of depression in women caregivers (Liu et al. 2024). Global research has also shown caregivers frequently experience a steady decline in life satisfaction, increased loneliness, and symptoms of depression, and women experience these declines to a greater extent than men (Krämer and Bleidorn 2024; Szebehely and Meagher 2018; World Health Organization [WHO], 2024). Additionally, caregiving often competes with other types of social activities and self-care, placing caregivers at a greater risk of burnout (Schulz and Eden 2016).

The COVID-19 pandemic magnified these issues globally. In the United States (U.S.) and other countries, caregivers also reported increased use of maladaptive coping behaviors (e.g., alcohol and substance use) to manage stress during the pandemic (Varma et al. 2024; Wong et al. 2023). In women caregivers, such behaviors are often associated with increased stress and disrupted sleep that is sometimes influenced by cultural norms surrounding alcohol use (Ayyala-Somayajula et al. 2024). In caregivers already exposed to heightened and chronic stress, drinking may further perpetuate psychological distress and not act as a single risk factor. Many high-income countries have demonstrated associations between caregiving stress, financial strain, and heightened alcohol consumption among women (Wadd and Galvani 2014; Szebehely and Meagher 2018). In low-resource settings, economic strain is often more extreme due to the limited institutional caregiving support, which further impacts mental health outcomes (WHO, 2024). Many caregivers reduce work hours or exit the workforce, leading to lost income, suspended retirement savings, and increased poverty risk (NAC & AARP, 2020; TIAA Institute 2023). Loss of income can increase a person’s risk of financial insecurity, which has been linked to worse mental health outcomes (Bialowolski et al. 2021; Ettman et al. 2023; Kiely et al. 2015).

Financial hardship and caregiver burden are among the most consistent predictors of depression. (Barakat et al. 2025; Gill et al. 2025). Few studies have explored the contribution of these variables to alcohol misuse among women caregivers, especially when alcohol misuse is assessed dichotomously instead of as a count-based variable (Barakat et al. 2025; Gill et al. 2025). Additionally, much of the existing research uses dichotomous measures of alcohol consumption, which fail to capture cyclical and frequency-based drinking patterns that represent caregiving stressors (Gill et al. 2025). Financial hardship has consistently been shown to predict depression (Bialowolski et al. 2021; Ettman et al. 2023; Lalani et al. 2025). Caregivers facing chronic financial distress may engage in maladaptive coping behavior, including increased alcohol use as a coping mechanism to alleviate emotional distress.

This study examined whether alcohol misuse moderates the relationship between financial hardship and mental health outcomes. Prior studies have shown an association between caregiving stress, financial hardship, and increased alcohol use (Strzelecki et al. 2022; Varma et al. 2024; Wadd and Galvani 2014). However, few studies (Gill et al. 2025; Keyes et al. 2011; Shuai et al. 2022) have measured whether alcohol use differentially predicts mental health among people facing economic hardship. In fact, alcohol misuse has frequently been conceptualized as a mediator of stress-related mental health symptoms. In this case, however, caregiving is a chronic structural stressor which may not be fully accounted for by alcohol use alone. Alcohol misuse may, instead, condition the experience of psychological distress in response to economic strain, and moderation is therefore conceptually plausible. Thus, the present study aimed to examine alcohol misuse as a moderator of the relationship between financial hardship and mental health outcomes among U.S. women caregivers. This approach considers both the presence and frequency of alcohol misuse and contextualizes the results in caregiving trends.

## Methods

### Sample

Data for the current study came from the 2023 Behavioral Risk Factor Surveillance System (BRFSS) survey. The BRFSS is a population-based health survey that collects data on health-related risk behaviors, chronic conditions, and preventive services of noninstitutionalized individuals (Centers for Disease Control and Prevention [CDC], 2023). The analytical sample consisted of 4,212 U.S. women caregivers who were 18 years or older, answered the three financial hardship items, and provided responses to the two mental health outcomes. The current study was deemed exempt by the institutional review board, as it used a publicly available, de-identifiable dataset.

### Measures

This study had two mental health outcomes. Depression diagnosis was measured by, “Ever been told that you had a depressive disorder, including depression, major depression, dysthymia, or minor depression?” Responses were yes and no. All variables were coded as binary responses unless noted. Number of days with poor mental health was measured by, “Now, thinking about your mental health, which includes stress, depression, and problems with emotions, for how many days during the past 30 days was your mental health not good?”

Financial hardship resulting in food insecurity was measured by, “During the past 12 months, how often did the food that you bought not last, and you didn’t have money to get more?” Responses included: always, usually, sometimes, rarely, and never. The responses were reverse coded to indicate increasing food insecurity. General financial hardship was measured by, “During the last 12 months, was there a time when you were not able to pay your mortgage, rent, or utility bills?” Financial hardship resulting in essential services being at risk was measured by, “During the last 12 months was there a time when an electric, gas, oil, or water company threatened to shut off services?” The calculated item, which identified whether participants were binge drinkers, was used as a proxy for alcohol misuse with responses yes and no. Alcohol misuse frequency was measured by, “Considering all types of alcoholic beverages, how many times during the past 30 days did you have 4 or more drinks on occasion?”

Missing and refused responses for alcohol misuse frequency were treated as ‘zero’ responses when participants answered the alcohol misuse item. To evaluate whether missing data could have introduced bias, individuals who provided data on all study variables were compared with individuals who did not provide complete data on all study variables of interest (i.e., demographic information and key study variables). Because the amount of missingness in this study was low, it was not necessary to use sophisticated approaches such as multiple imputation or full information maximum likelihood. Missingness was not significantly associated with age, income, education, the financial strain or health hardship indicators, or mental health outcomes (all *p* >.05).

Moderation was assessed by adding interaction terms between the financial hardship measures and the two alcohol misuse measures to these models. The interaction terms were created by multiplying each of the financial hardship measures by alcohol misuse status and alcohol misuse frequency. Significant interaction terms were interpreted as an indication that alcohol misuse moderated the relationship between financial hardship and mental health.

Covariates included demographics and caregiving information. Demographics included age, race/ethnicity, educational level, annual household income, marital status, urban-rural status, and health insurance status. Caregiving information included: relationship to care recipient, medical condition of care recipient, length of time of provider care, intensity of care, managing personal care, and managing household tasks. Missing or refused responses were recoded to the most conservative category for income, marital status, health insurance status, and all caregiving information variables.

### Data analysis

To account for the BRFSS’s complex survey design and weighting, data were analyzed using IBM SPSS 23.0 Complex Samples module. To examine the demographics of participants according to the depression diagnosis, the χ^2^ test was used for categorical variables and the t-test for continuous variables (Table 1). Multivariable logistic regression analyses were performed to examine the associations between financial hardship indicators and depression diagnosis (Table 2). Multivariable linear regression analyses were performed to examine the associations between financial hardship indicators and the number of days with poor mental health (Table 3). Moderation results are shown in separate columns for ease of interpretation using interaction terms.

**Table 1:** The characteristics of U.S. Women caregivers by their depression diagnosis, 2023 behavioral risk factor surveillance system (*N* = 4,212)

	*Depression diagnosis*			
Characteristics	No (*n* = 2,962) n (%)	Yes (*n* = 1,250) n (%)	*χ*^2^	*p*-value
Number of days with poor mental health	4.22^a^ (0.23^b^)	13.06^a^ (0.49^b^)	17.70^c^	< 0.001
Financial hardship that leads to food insecurity			195.71	< 0.001
Never	2,268 (72.2)	713 (51.7)		
Rarely	310 (12.1)	189 (17.0)		
Sometimes	240 (10.4)	198 (17.8)		
Usually	61 (2.6)	65 (6.7)		
Always	83 (2.7)	85 (6.8)		
General financial hardship			147.35	< 0.001
No	2,684 (89.0)	960 (74.5)		
Yes	278 (11.0)	290 (25.5)		
Financial hardship that leads to essential services being at risk			102.29	< 0.001
No	2,764 (91.4)	1,053 (80.6)		
Yes	198 (8.6)	197 (19.4)		
Alcohol misuse			0.37	0.679
No	2,717 (90.4)	1,116 (89.8)		
Yes	245 (9.6)	134 (10.2)		
Alcohol misuse frequency in last 30 days	0.34^a^ (0.04^b^)	0.63^a^ (0.12^b^)	3.29	0.001
Age			122.65	< 0.001
18–24	76 (6.0)	62 (9.2)		
25–34	144 (9.2)	120 (15.3)		
35–44	292 (14.8)	182 (19.2)		
45–54	447 (17.0)	248 (19.8)		
55–64	634 (21.8)	271 (18.2)		
65 or older	1,369 (31.2)	367 (18.3)		
Race/ethnicity			77.53	< 0.001
Non-Hispanic White	1,914 (57.9)	892 (65.7)		
Non-Hispanic Black	226 (14.2)	69 (10.0)		
Non-Hispanic Asian	199 (5.3)	39 (1.8)		
Non-Hispanic American Indian/Alaskan Native	79 (2.3)	29 (2.4)		
Hispanic	251 (14.6)	104 (10.9)		
Non-Hispanic other race	293 (5.7)	117 (9.3)		
Educational level			23.50	0.041
Did not graduate high school	108 (7.1)	67 (9.3)		
Graduated high school	628 (27.1)	287 (24.7)		
Attended college or technical school	943 (35.7)	451 (40.9)		
Graduated from college or technical school	1,275 (30.0)	441 (24.9)		
Do not know/Not sure	8 (0.1)	4 (0.2)		
Income			60.67	< 0.001
Less than $15,000	583 (21.4)	290 (26.6)		
$15,000 - $24,999	220 (7.5)	138 (9.2)		
$25,000 - $34,999	286 (9.7)	156 (12.8)		
$35,000 - $49,999	411 (12.7)	184 (13.9)		
$50,000 - $99,999	820 (26.5)	300 (23.4)		
$100,000 - $199,999	512 (17.3)	156 (11.6)		
$200,000 or more	130 (4.9)	26 (2.4)		
Marital status			120.84	< 0.001
Married	1,792 (59.2)	590 (46.6)		
Divorced	362 (11.0)	219 (13.7)		
Widowed	359 (8.3)	119 (8.1)		
Separated	45 (2.0)	62 (5.6)		
Never married	337 (17.4)	201 (19.5)		
A member of an unmarried couple	67 (2.2)	59 (6.5)		
Urban-rural status			1.06	0.475
Rural	326 (6.9)	146 (7.8)		
Urban	2,636 (93.1)	1,104 (92.2)		
Health insurance status			0.73	0.639
Do not have health insurance	182 (7.9)	74 (8.7)		
Have health insurance	2,780 (92.1)	1,176 (91.3)		
Relationship to care recipient			23.75	0.040
Spouse/partner	652 (17.2)	245 (14.4)		
Parent/parent-in-law	867 (33.4)	370 (31.5)		
Child/grandchild	338 (12.7)	186 (17.6)		
Other relative	541 (20.8)	224 (22.1)		
Nonrelatives	564 (20.8)	225 (14.4)		
Medical condition of care recipient			3.09	0.710
Cancer	209 (7.2)	89 (6.2)		
Dementia	352 (11.4)	137 (10.3)		
Cardiovascular diseases	418 (11.4)	173 (13.9)		
Other	1,983 (67.8)	851 (69.6)		
Length of time provider care			14.53	0.052
Less than 6 months	920 (27.9)	346 (27.3)		
6 months to less than 5 years	1,189 (41.7)	463 (36.8)		
5 or more years	853 (30.5)	441 (36.0)		
Intensity of care (hours per week)			7.21	0.404
Up to 8	1,634 (52.0)	635 (48.9)		
9–19	369 (12.8)	159 (12.4)		
20–39	332 (11.7)	165 (14.3)		
40 or more	627 (23.6)	291 (24.4)		
Managing personal care, past 30 days			16.18	0.009
No	1,481 (45.6)	548 (39.0)		
Yes	1,481 (54.4)	702 (61.0)		
Managing household tasks			17.40	0.003
No	624 (18.9)	223 (13.7)		
Yes	2,338 (81.1)	1,027 (86.3)		

Note. Percentages are weighted. ^a^Weighted mean; ^b^Weighted standard error; ^c^*t*-statistic

**Table 2:** The association between financial hardship indicators and depression diagnosis of U.S. women caregivers, 2023 Behavioral Risk Factor Surveillance System (N = 4,212)

	Total sample		Alcohol misuse vs. no misuse		Alcohol misuse frequency	
Predictors	OR (95% CI)	aOR (95% CI)†	OR (95% CI)	aOR (95% CI)†	OR (95% CI)	aOR (95% CI)†
Financial hardship that leads to food insecurity (5-point scale)	1.31 (1.18, 1.46)***	1.23 (1.10, 1.38)^***^	0.91 (0.73, 1.13)	0.85 (0.68, 1.07)	1.03 (1.01, 1.05)**	1.02 (1.003, 1.04)^*^
General financial hardship						
No	Reference	Reference	Reference	Reference	Reference	Reference
Yes	1.64 (1.16, 2.32)^**^	1.56 (1.10, 2.20)*	4.45 (1.73, 11.44)^**^	4.09 (1.59, 10.49)^**^	1.38 (1.17, 1.63)^***^	1.34 (1.15, 1.57)^***^
Financial hardship that leads to essential services being at risk						
No	Reference	Reference	Reference	Reference	Reference	Reference
Yes	1.36 (0.92, 1.99)	1.29 (0.88, 1.91)	0.69 (0.23, 2.05)	0.70 (0.24, 2.04)	0.78 (0.66, 0.92)^**^	0.78 (0.66, 0.91)^**^

Note. OR, odds ratio; CI, confidence interval; aOR, adjusted odds ratio

* *p* <.05; ** *p* <.01; *** *p* <.001

Financial hardship that leads to food insecurity is a 5-point scale with the following responses: never, rarely, sometimes, usually, and always

†After controlling for age, race/ethnicity, educational level, income, marital status, urban-rural status, health insurance status, relationship to care recipient, medical condition of care recipient, length of time of provider care, intensity of care, managing personal care, and managing household tasks

**Table 3:** The association between financial hardship indicators and the number of days with poor mental health of U.S. Women caregivers, 2023 behavioral risk factor surveillance system (*N* = 4,212)

	*Total sample*		*Alcohol misuse vs. no misuse*		*Alcohol misuse frequency*	
Predictors	aB (SE)	aB (SE)†	aB (SE)	aB (SE)†	aB (SE)	aB (SE)†
Financial hardship that leads to food insecurity (5-point scale)	1.78 (0.29)^***^	1.37 (0.29)^***^	1.02 (0.57)	0.72(0.58)	0.22 (0.04)^***^	0.19 (0.05)^***^
General financial hardship						
No	Reference	Reference	Reference	Reference	Reference	Reference
Yes	2.53 (0.98)^*^	2.15 (0.94)^*^	2.49 (3.05)	1.60 (2.86)	0.14 (0.18)	0.14 (0.21)
Financial hardship that leads to essential services being at risk						
No	Reference	Reference	Reference	Reference	Reference	Reference
Yes	3.70 (1.06)^***^	3.29 (1.04)^**^	4.97 (3.17)	4.52 (3.07)	0.19 (0.23)	0.11 (0.26)

Note. aB, adjusted unstandardized regression coefficient; SE, standard error.

* *p* <.05; ** *p* <.01; *** *p* <.001.

Financial hardship that leads to food insecurity is a 5-point scale with the following responses: never, rarely, sometimes, usually, and always.

†After controlling for age, race/ethnicity, educational level, income, marital status, urban-rural status, health insurance status, relationship to care recipient, medical condition of care recipient, length of time of provider care, intensity of care, managing personal care, and managing household tasks

## Results

Table 1 presents the descriptive statistics for women caregivers from the U.S. categorized by their depression diagnosis. Women caregivers with a depression diagnosis experienced significantly more days of poor mental health and were more likely to report all indicators of financial hardship, most notably food insecurity. Depression was also more common among those with lower income and more caregiving responsibilities related to both personal care and household activities. While the prevalence of alcohol misuse did not differ by depression status, frequency of misuse was significantly higher for those with depression.

Women caregivers diagnosed with depression reported significantly poorer mental health, averaging 13.06 days of poor mental health in the past month compared to 4.22 days without depression (*p* <.001). Financial hardship resulting in food insecurity (“always” 6.8% depression vs. 2.7% no depression; *p* <.001), general financial hardship (25.5% depression vs. 11.0% no depression; *p* <.001), and trouble affording essential services (19.4% depression vs. 8.6% no depression; *p* <.001) were more commonly reported by women caregivers with depression. Women caregivers with depression were more likely to have a college education (71.4% vs. 28.6%, *p* =.041) and have earnings below $35,000, indicating socioeconomic differences (48.6% vs. 38.6%; *p* <.001). Depression was more common among those who were never married (*p* <.001). Non-Hispanic Whites had a larger percentage of women suffering from depression (65.7% vs. 57.9%), indicating a significant association between race/ethnicity and depression (*p* <.001). Depression was associated with an increase in the prevalence of caregiving responsibilities, including coordination of personal care (*p* =.009) and domestic duties (*p* =.003). Finally, there was a significant association between the relationship between the person receiving care and depression (*p* =.040).

### Depression diagnosis

Table 2 shows the associations between the financial hardship indicators and depression diagnosis among U.S. women caregivers. Table 2 indicates that financial hardship, in particular food insecurity and general financial strain, was consistently and positively associated with higher odds of depression among women caregivers, even after adjustment for sociodemographic and caregiving factors. Alcohol misuse as a binary status was not independently associated with depression, but higher frequency of misuse strengthened the association between food insecurity and depression.

Most indicators of financial hardship and depression diagnosis were still significant even when accounting for alcohol misuse and misuse frequency. In the adjusted models, going through financial hardship that resulted in food insecurity was associated with a 23% higher chance of being diagnosed with depression (adjusted odds ratio [aOR]: 1.23; 95% confidence interval [CI]: 1.10, 1.38). General financial hardship increased the likelihood of depression in women caregivers by 56% (aOR: 1.56; 95% CI: 1.10, 2.20). Caregivers that misused alcohol and faced general financial hardship were 309% more likely to report depression than those who neither misused alcohol nor faced general financial hardship (aOR: 4.09; 95% CI: 1.59, 10.49).

For each increase in the frequency of alcohol misuse, the likelihood of being diagnosed with depression increased by 2% for those experiencing financial hardship leading to food insecurity (aOR: 1.02; 95% CI: 1.003, 1.07). Moreover, caregivers who experienced general financial hardship had a 34% greater likelihood of having a depression diagnosis than caregivers who did not have a general financial hardship (aOR: 1.34; 95% CI: 1.15, 1.57). Furthermore, caregivers who experienced financial hardship that jeopardized essential services had a 22% lower likelihood of being diagnosed with depression compared to caregivers who did not experience financial hardship that jeopardized essential services (aOR: 0.78; 95% CI: 0.66, 0.91). Thus, financial hardship that jeopardized essential services was inversely associated with depression diagnosis in adjusted models.

### Number of days with poor mental health

Table 3 presents the associations between the financial hardship indicators and the number of poor mental health days among U.S. women caregivers. As shown in Table 3, financial hardship was associated with more poor mental health days, and the highest increases were seen among caregivers with food insecurity and hardship related to essential services. The impact of alcohol misuse frequency on poor mental health days among food-insecure caregivers was also stronger than the main effect of alcohol misuse status.

There was a significant association between several indicators of financial hardship and the number of days with poor mental health when moderated by alcohol misuse and alcohol misuse frequency, respectively. Caregivers facing financial hardship resulting in food insecurity reported 1.37 more days of poor mental health (adjusted unstandardized regression coefficient [aB]: 1.37; standard error (SE): 0.29; *p* <.001), and caregivers with general money problems had 2.15 more days (aB: 2.15; SE: 0.94; *p* <.05). Additionally, those struggling with financial issues related to essential services had 3.29 more days of poor mental health (aB: 3.29; SE: 1.04; *p* <.01). For caregivers experiencing financial hardship due to food insecurity, each additional instance of alcohol misuse was associated with an additional 0.19 days of poor mental health (aB: 0.19; SE: 0.05; *p* <.001).

## Discussion

This study represents an important first step in identifying the interplay between financial and behavioral distress in caregiver mental health among U.S. women. The binary alcohol misuse outcome did not have a significant independent relationship to negative mental health outcomes. However, a novel insight from the current study is the reported frequency of alcohol misuse had a significant modifying effect on poor mental health outcomes, particularly in the context of food insecurity. These findings indicate that the frequency of alcohol misuse may exacerbate the negative mental health consequences of material deprivation, with financial strain having a more pronounced impact on psychological distress among caregivers who reported higher levels of alcohol consumption (Møller et al. 2019; Shuai et al. 2022). This relationship represents a new addition to the literature on caregiver stress and behavioral health risk.

Our bivariate findings in Table 1 indicated that younger caregivers were disproportionately represented among those who reported depression. Caregiving during earlier stages of the life course often coincides with economic instability, and competing role demands such as childrearing and early career development, which may amplify psychological strain. In addition, caregiving roles may be less anticipated earlier in adulthood, and the unexpected emergence of caregiving responsibilities may intensify distress by disrupting planned life trajectories and limiting available coping resources. Additionally, the bivariate finding of a higher percentage of non-Hispanic White caregivers with reported depression diagnoses should be made with caution because diagnosis is a proxy for care and help-seeking and may not be a true reflection of underlying psychological distress across racial and ethnic groups. Prior research suggests that racial and ethnic minority caregivers often face structural barriers to mental health care and may therefore be less likely to receive formal diagnoses despite experiencing comparable or greater levels of distress (Ervin et al. 2022; NAC & AARP, 2020). Counterintuitively, the finding of higher depression diagnosis among more highly educated caregivers may be due to more opportunities for and access to mental health services and awareness of symptoms and means of diagnosis, rather than actual higher prevalence of distress. Previous research demonstrated that financial hardship or strain contributes to mental health issues (Bialowolski et al. 2021; Kiely et al. 2015; Ryu and Fan 2023). The current findings support previous literature, including studies by Ettman et al. (2023) and Lalani et al. (2025), which indicate that financial insecurity is a significant driving factor for mental health outcomes, especially among women who provide unpaid care. Gendered differences in caregiving can increase the risk of mental health illness, with women more likely to experience these conditions, an impact worsened by socioeconomic factors (Bueno and Chase 2023; Zwar et al. 2023). Of the three measures of financial hardship, being food insecure predicted higher levels of depression and psychological distress. This finding aligns with previous literature, such as Xu et al. (2021), which has shown that having a low income is associated with increased caregiver burden. As evidenced in the present study, women caregivers with financial hardship and food insecurities are more susceptible to mental health challenges, including alcohol misuse.

Unexpectedly, in adjusted models, financial hardship that had been severe enough to cause concerns about being able to pay for essential services at the time of data collection was associated with lower odds of a reported diagnosis of depression. It is unlikely that this finding represents a true protective effect of such hardship, but instead may reflect a selection or detection bias. Caregivers whose lives are in an acute material crisis may understandably focus on day-to-day survival needs and not seek or receive a depression diagnosis, resulting in an apparent lower distress. The association may also reflect confounding by service engagement, with caregivers who approach emergency assistance organizations (e.g., social service or community organizations) as a function of acute hardship experiencing temporary buffering of psychological distress or promotion of problem-focused coping. Financial hardship may also operate nonlinearly, with greater material deprivation leading to altered coping behaviors or reporting patterns, rather than decreased depression symptoms. Finally, this association may be explained in part by unmeasured factors such as informal support systems, local community resources, or sociocultural attitudes toward disclosure of distress.

A key finding is that alcohol misuse was not an independent risk factor for depression. Instead, frequency of misuse was associated with worse mental health days, particularly among food-insecure women. This pattern may be reflective of the role of alcohol as a facilitator of distressful thoughts and behaviors, as suggested by theories of maladaptive coping (Lazarus and Folkman 1984; Matei-Mitacu et al. 2024). This finding is in line with research showing an increase in alcohol use among women caregivers (Ayyala-Somayajula et al. 2024; Varma et al. 2024) and more broadly highlights the fluidity of sociocultural norms around alcohol use in chronic caregiving stress. This is an important consideration when assessing the risks of alcohol misuse in caregiver populations; that is, it may not be sufficient as a single marker of behavioral health risk. Our findings suggest a need to move away from dichotomous models of substance use screening and instead emphasize a more detailed examination of use patterns and the structural conditions that underlie them.

There existed a lack of an association between alcohol misuse and mental health outcomes, incongruent with the existing literature, which clearly links alcohol use with depression among caregivers (Strzelecki et al. 2022) and women more broadly (Fredman Stein et al. 2022; Meshesha et al., 2020; McCaul et al. 2019). However, prior research often relied on dichotomous measures of alcohol use or misuse, which does not capture variability in drinking or context of use. The present study’s ability to separate the presence versus frequency of alcohol misuse provided the needed specificity to uncover that alcohol use is not a unique predictor of depression but is associated with greater psychological distress among women facing other structural stressors, in this case, food insecurity. provides more evidence regarding the role of alcohol misuse, as a conditional or amplifying mechanism rather than a distinct risk factor.

### Implications for policy, practice, and global relevance

The findings from this research provide valuable insight for public health, caregiver support systems, and international caregiving policy. While U.S. data was used for analysis, the findings linking structural economic vulnerability and maladaptive alcohol use to declining mental health are relevant to geopolitical contexts. Worldwide, unpaid caregiving is a gendered labor activity that contributes to existing inequities in income, healthcare access, and social support. In the U.S., screening processes for caregivers should include assessment for mental health and substance use as well as financial strain. These recommendations overlap with caregiver-targeted interventions and services that are currently provided to caregivers and pair financial assistance with mental health and behavioral strategies. For example, caregiver interventions that have been tested in rigorous research settings and rely on group education and skills-building to connect caregivers to more far-reaching community services include the Resources for Enhancing Alzheimer’s Caregiver Health intervention and the National Family Caregiver Support Program, both of which have a positive impact on caregiver mental health (Feinberg et al. 2011; NAC & AARP, 2020). Public health approaches such as integrated primary care screening and community health worker-led outreach have also been used in pilot approaches to screen for and support caregiver mental health alongside social determinants of health including food insecurity and financial hardship (Billioux et al. 2017; Alley et al. 2016). Though these programs differ in their scope and the populations they serve, they represent feasible approaches for implementing the trauma-informed, harm-reduction, and financially responsive caregiver support recommended. Caregiver screening should be a collaboration between primary care providers, community health workers, and social service professionals who should be trained in the additive effects of economic and behavioral stress and acknowledge the gendered dimensions of caregiving that often place a heavier burden on women.

Additionally, the present results are consistent with UNICEF’s ‘Caring for the Caregiver’ initiative (2024), which calls for attending to caregivers’ mental health as a public health issue. In countries with strong social protection measures such as paid caregiving leave, women caregivers continue to experience feelings of loneliness, role overload, and depression (Szebehely and Meagher 2018). In low-resource countries, where families provide the most care and institutional support is minimal, disparities in caregiving can worsen the burden. Alcohol misuse as a coping strategy is a transnational issue; cultural norms may range from high per capita consumption in some Eastern European countries to restrictive norms against alcohol consumption in Middle Eastern contexts. However, Australian, British, and Canadian studies have identified a link between the stress of caregiving in addition to financial strain and increased alcohol consumption in women (Ervin et al. 2022; Wadd and Galvani 2014). The use of a frequency-based assessment in this study may present an internationally translatable methodological refinement that can improve the cross-country comparability of alcohol misuse research.

In this study, intersectionality was used as a conceptual framework to situate caregivers’ experiences within intersecting systems of structural vulnerability, as opposed to a statistical test of intersectionality of identities. In the present analyses, intersectional interactions were not explicitly modeled, but the resulting patterns for age, race, economic hardship, and alcohol misuse should be considered in the context of broader social systems and constructs that encompass access to resources, health systems, and social location. This framing allows for an explanation of diagnosis, service access, and exposure to risk without speculating outside of the data. Since depression diagnosis is an indicator of health systems of contact, differences in access and trust may also determine which caregivers are formally diagnosed in population-based surveys.

In addition, an intersectional approach is critical for understanding vulnerability among caregivers. Caregiving burdens in different contexts are influenced by intersecting identities, such as migration status, race and ethnicity, disability, and sexual orientation. For example, migrant women who are caregivers in the European Union have experienced more exploitative working conditions and limited access to social benefits and services (Hussein 2022). Indigenous women caregivers in Latin America have also faced intersectional disadvantages, particularly due to geographic isolation, poverty, and a gendered inequitable health system (Zuluaga et al. 2025). The same structural disadvantages have impacted numerous women caregivers throughout the U.S. (Ervin et al. 2022). Ultimately, our findings suggest trauma-informed, harm-reduction interventions addressing financial hardship and maladaptive coping strategies. These findings could also be used by international organizations such as the WHO, the ILO, or UN Women to support the development of global caregiver well-being indicators and gender-responsive social protection advocacy. Similar approaches have been adapted in the global arena into the design of caregiver wellbeing pilots embedded in primary care and social protection systems in international settings with a specific focus on community-based care and task-sharing models.

### Strengths and contributions

This study’s strengths are multifaceted. First, the use of a nationally representative sample allowed for generalizability of findings to the general U.S. population. The focus on the experiences of a group of U.S. women caregivers brings this population to the forefront, as women caregivers are understudied in quantitative behavioral health research. The positioning of health behaviors in a relationship to intersecting multi-dimensional structural forces extended the existing dichotomous research. Finally, by operationalizing alcohol misuse as a continuum of frequency, this study contributes to the methodological rigor and support for future studies to move beyond simplistic measures.

### Limitations

Despite these strengths, the study has several limitations. The study’s cross-sectional design prevents drawing causal inference regarding mediation, which in turn raises questions regarding the directionality between alcohol use and psychological distress. Thus, alcohol misuse frequency is a contextual factor rather than a mediator in that condition, so it should be noted that the condition does not transmit the effects of financial hardship on mental health. It is unclear whether alcohol use exacerbates mental health issues or whether women turn to alcohol in response to worsening mental health problems as a maladaptive coping mechanism. Additionally, the use of self-reported measures may introduce recall and prevarication bias. While some participants had ‘missing’ responses, the number was low, and complete case analyses had robust results. Analyses comparing participants with complete and missing data indicated low risk of bias. The BRFSS dataset also lacks information on emotional and relational caregiving dynamics—factors that likely mediate both alcohol use and mental health outcomes. Moreover, another limitation is that we included missing and refused responses for certain variables when it was appropriate for completeness and sample size considerations. However, the number of missing and refused responses was small, and so the limitation is minimal and not likely to affect overall findings.

### Future research directions

Future studies should adopt longitudinal designs to better assess causal pathways and change over time. The integration of more fine-grained markers, such as emotional labor, relationship strain, and caregiver satisfaction, will allow for a more comprehensive assessment of the caregiving experience. In addition, intersectional analyses are crucial. It is possible to develop more equitable policies and interventions by understanding how aspects of race, class, disability, and sexual orientation shape outcomes of caregiving and behavioral health. It is important to include qualitative and mixed-methods research to effectively capture the lived experiences of caregivers and provide context for the patterns solely observed in quantitative studies.

## Conclusion

This study highlights the importance of the intersection between financial hardship, particularly food insecurity, and the frequency of alcohol misuse being associated with women caregivers’ mental health, a finding with implications beyond the U.S. Although this study uses nationally representative data from the U.S., it highlights underlying mechanisms that are applicable in most—if not all—contexts: structural financial vulnerabilities, gendered expectations for caregiving responsibilities, and substance use as a maladaptive coping strategy. The alcohol use frequency measure used in this study is a useful contribution to better capture behavioral coping mechanisms under chronic stress. The framing of this study as it relates to caregivers’ well-being within both structural and feminist health perspectives demonstrates the importance of structural and intersecting identities in terms of risk exposure and resources.

Our results can also be used to inform trauma-informed, harm-reduction approaches that screen for and mitigate material and behavioral risk factors, and that are adaptable to multiple health systems and service delivery contexts. In higher-income countries, these approaches could be built into long-term care and routine caregiver assessments; in low-resource environments, they may be a critical part of community-based outreach and primary care services. Given the increasing global need for caregiving and ongoing social and economic inequities, researchers must use comparative, cross-national designs to identify how policy, cultural environments, and health system designs affect caregiver mental health.

## Data Availability

The dataset analyzed during this study is publicly available at the CDC’s website, under the 2023 annual survey file link at https://www.cdc.gov/brfss/annual\_data/annual\_2023.html.

## References

[CR1] Alley DE, Asomugha CN, Conway PH, Sanghavi DM (2016) Accountable health communities—addressing social needs through medicare and medicaid. N Engl J Med 374(1):8–11. 10.1056/NEJMp1512532 26731305 10.1056/NEJMp1512532

[CR2] Ayyala-Somayajula D, Dodge JL, Leventhal AM, Terrault NA, Lee BP (2024) Trends in alcohol use after the COVID-19 pandemic: a national cross-sectional study. Ann Intern Med 178(1):139–142. 10.7326/ANNALS-24-02157 39527820 10.7326/ANNALS-24-02157

[CR3] Barakat Z, Al-Sabbah H, Badrasawi M, Abu-Al-Samen M, Hawamdeh S (2025) Examining burden among caregivers of community-dwelling older adults with chronic diseases: a cross-sectional study. Sci Rep 15(1):1269. 10.1038/s41598-025-05626-5 40594243 10.1038/s41598-025-05626-5 PMC12218568

[CR4] Basu S, Berkowitz SA (2022) Unpaid family caregiving—the next frontier of gender equity in a postpandemic future. JAMA Health Forum 3(3):e220642. 10.1001/jamahealthforum.2022.0642 10.1001/jamahealthforum.2023.1310 37294582

[CR5] Bialowolski P, Weziak-Bialowolska D, Lee MT, Chen Y, VanderWeele TJ, McNeely E (2021) The role of financial conditions for physical and mental health. Evidence from a longitudinal survey and insurance claims data. Soc Sci Med 281:114041. 10.1016/j.socscimed.2021.114041 34087548 10.1016/j.socscimed.2021.114041

[CR6] Billioux A, Verlander K, Anthony S, Alley D (2017) *Standardized screening for health-related social needs in clinical settings: The Accountable Health Communities Screening Tool.* National Academy of Medicine. https://nam.edu/wp-content/uploads/2017/05/Standardized-Screening-for-Health-Related-Social-Needs-in-Clinical-Settings.pdf

[CR7] Bueno MV, Chase JAD (2023) Gender differences in adverse psychosocial outcomes among family caregivers: a systematic review. West J Nurs Res 45(1):78–92. 10.1177/01939459221099672 35614567 10.1177/01939459221099672

[CR8] Centers for Disease Control and Prevention (2023) *BRFSS overview*. U.S. Department of Health and Human Services. https://www.cdc.gov/brfss/annual_data/2023/pdf/Overview_2023-508.pdf

[CR9] Ervin J, Taouk Y, Alfonzo LF, Peasgood T, King T (2022) Longitudinal association between informal unpaid caregiving and mental health amongst working age adults in high-income OECD countries: a systematic review. EClinicalMedicine. 10.1016/j.eclinm.2022.101711 36353526 10.1016/j.eclinm.2022.101711 PMC9637877

[CR10] Ettman CK, Fan AY, Philips AP, Adam GP, Ringlein G, Clark MA, Wilson IB, Vivier PM, Galea S (2023) Financial strain and depression in the U.S.: a scoping review. Transl Psychiatry 13(1):168. 10.1038/s41398-023-02460-z 37179345 10.1038/s41398-023-02460-z PMC10182750

[CR11] Feinberg LF, Reinhard SC, Houser A, Choula R (2011) *Valuing the invaluable: 2011 update—The growing contributions and costs of family caregiving.* AARP Public Policy Institute. https://collections.nlm.nih.gov/master/borndig/101565021/i51-caregiving.pdf

[CR12] Fredman Stein K, Allen JL, Robinson R, Smith C, Sawyer K, Taylor G (2022) Do interventions principally targeting excessive alcohol use in young people improve depression symptoms? A systematic review and meta-analysis. BMC Psychiatry 22(1). 10.1186/s12888-022-04006-x 10.1186/s12888-022-04006-x PMC9214998 35729518

[CR13] Gill A, Clum G, Molina P, Welsh D, Ferguson T, Theall KP (2025) Life course stressors, latent coping strategies, alcohol use, and adherence among people with HIV. AIDS Behav 29(2):589–599. 10.1007/s10461-024-04541-6 39546146 10.1007/s10461-024-04541-6 PMC11814058

[CR14] Hussein S (2022) The global demand for migrant care workers: drivers and implications on migrants’ wellbeing. Sustainability 14(17):10612. 10.3390/su141710612

[CR15] International Labour Organization (2018) *Care work and care jobs for the future of decent work*. https://www.ilo.org/resource/news/ilo-women-do-4-times-more-unpaid-care-work-men-asia-and-pacific

[CR16] Keyes KM, Hatzenbuehler ML, Hasin DS (2011) Stressful life experiences, alcohol consumption, and alcohol use disorders: the epidemiologic evidence for four main types of stressors. Psychopharmacology 218(1):1–17. 10.1007/s00213-011-2236-1 21373787 10.1007/s00213-011-2236-1 PMC3755727

[CR17] Kiely KM, Leach LS, Olesen SC, Butterworth P (2015) How financial hardship is associated with the onset of mental health problems over time. Soc Psychiatry Psychiatr Epidemiol 50(6):909–918. 10.1007/s00127-015-1027-0 25683473 10.1007/s00127-015-1027-0

[CR18] Krämer MD, Bleidorn W (2024) The well-being costs of informal caregiving. Psychol Sci 35(12):1382–1394. 10.1177/09567976241279203 39585689 10.1177/09567976241279203

[CR19] Lalani N, Osei EA, Yang S, Katare B, Wagle S (2025) Financial health and well-being of rural female caregivers of older adults with chronic illnesses. BMC Geriatr 25(1):239. 10.1186/s12877-025-05853-5 40211160 10.1186/s12877-025-05853-5 PMC11984026

[CR20] Lazarus RS, Folkman S (1984) Stress, appraisal, and coping. Springer Publishing Company

[CR21] Liu Y, Hughes MC, Wang H (2024) Financial strain, health behaviors, and psychological well-being of family caregivers of older adults during the COVID-19 pandemic. PEC Innovation 4:100290. 10.1016/j.pecinn.2024.100290 38799257 10.1016/j.pecinn.2024.100290 PMC11127198

[CR22] Matei-Mitacu LM, Huțul TD, Karner-Huțuleac A, Huțul A, Dobria CA (2024) The role of alcohol consumption motives in the relationships between psychological distress, emotional dysregulation, and problematic alcohol consumption. A mediation model. Curr Psychol 43(48):36831–36845. 10.1007/s12144-024-07111-0

[CR23] McCaul ME, Roach D, Hasin DS, Weisner C, Chang G, Sinha R (2019) Alcohol and women: a brief overview. Alcohol Clin Exp Res 43(5):774. 10.1111/acer.13985 30779446 10.1111/acer.13985 PMC6502688

[CR24] Meshesha LZ, Aston ER, Teeters JB, Blevins CE, Battle CL, Marsh E, Feltus Sage, Stein Michael D., Abrantes AM (2020) Evaluating alcohol demand, craving, and depressive symptoms among women in alcohol treatment. Addict Behav 109:106475. 10.1016/j.addbeh.2020.106475 32480282 10.1016/j.addbeh.2020.106475 PMC7296463

[CR25] Møller SP, Pisinger VSC, Christensen AI, Tolstrup JS (2019) Socioeconomic position and alcohol-related harm in Danish adolescents. J Epidemiol Community Health 73:839–845. 10.1136/jech-2018-211634 31221897 10.1136/jech-2018-211634

[CR26] National Alliance for Caregiving & AARP (2020) Caregiving in the U.S. 2020. AARP Res. https://www.caregiving.org/research/caregiving-in-the-us-2020/

[CR27] Ryu S, Fan L (2023) The relationship between financial worries and psychological distress among US adults. J Fam Econ Iss 44(1):16–33. 10.1007/s10834-022-09820-9 10.1007/s10834-022-09820-9 PMC8806009 35125855

[CR28] Schulz R, Eden J (eds) (2016) Families caring for an aging America. National Academies 27905704

[CR29] Shuai R, Anker JJ, Bravo AJ, Kushner MG, Hogarth L (2022) Risk pathways contributing to the alcohol harm paradox: socioeconomic deprivation confers susceptibility to alcohol dependence via greater exposure to aversive experience, internalizing symptoms and drinking to cope. Frontiers in Behavioral Neuroscience 16:821693. 10.3389/fnbeh.2022.821693 35237137 10.3389/fnbeh.2022.821693 PMC8883115

[CR30] Strzelecki AM, Moloney ME, Brooks AT, Weafer J (2022) Alcohol use, sleep, and depression among family caregivers in the time of COVID-19. Alcohol 102:35–42. 10.1016/j.alcohol.2022.04.002 35500757 10.1016/j.alcohol.2022.04.002 PMC9052708

[CR31] Szebehely M, Meagher G (2018) Nordic eldercare – weak universalism becoming weaker? J Eur Soc Policy 28(3):294–308. 10.1177/0958928717735062

[CR32] TIAA Institute (2023) *Playing the long game: How longevity affects financial planning and family caregiving.* https://www.tiaa.org/content/dam/tiaa/institute/pdf/infographics/2023-10/tiaa-institute-financial-caregiving-infographic-november-2023.pdf

[CR33] UNICEF (2024) *Caring for the caregiver: Overview guide.* https://www.unicef.org/media/165006/file/UNICEF-caring-for-caregiver-overview-guide-2024.pdf

[CR34] Varma P, DePadilla L, Czeisler MÉ, Rohan EA, Weaver MD, Quan SF, Robbins R, Patel CG, Melillo S, Drane A, Winnay SS, Lane RI, Czeisler CA, Howard ME, Rajaratnam SMW, Matjasko JL (2024) Substance use and help seeking as coping behaviors among parents and unpaid caregivers of adults in the united States during the COVID-19 pandemic. Am J Drug Alcohol Abuse 50(6):851–863. 10.1080/00952990.2024.2394970 39436314 10.1080/00952990.2024.2394970 PMC11698632

[CR35] Wadd S, Galvani S (2014) Working with older people with alcohol problems: insight from specialist substance misuse professionals and their service users. Soc Work Educ 33(5):656–669. 10.1080/02615479.2014.919076

[CR36] Wong MC, Huang J, Wang HH, Yuan J, Xu W, Zheng ZJ, Association of Pacific Rim Universities (2023) Resilience level and its association with maladaptive coping behaviours in the COVID-19 pandemic: a global survey of the general populations. Globalization Health 19(1):1. 10.1186/s12992-022-00903-8 36597129 10.1186/s12992-022-00903-8 PMC9808687

[CR37] World Health Organization (2024) *WHO calls for urgent transformation of care and support systems for older people.* https://www.who.int/news/item/01-10-2024-who-calls-for-urgent-transformation-of-care-and-support-systems-for-older-people?

[CR38] Xu H, Kadambi S, Mohile SG, Yang S, Kehoe LA, Wells M, Loh KP (2021) Caregiving burden of informal caregivers of older adults with advanced cancer: the effects of rurality and education. J Geriatr Oncol 12(7):1015–1021. 10.1016/j.jgo.2021.04.002 33858803 10.1016/j.jgo.2021.04.002 PMC8429077

[CR39] Zuluaga LS, Montoya-Díaz JC, Scott CP, Bautista-Gomez MM (2025) Equitable community participation in health initiatives among Indigenous women in Latin America: a systematic review. BMJ Innov 11(2):98–106. 10.1136/bmjinnov-2024-001282

[CR40] Zwar L, König HH, Hajek A (2023) Gender differences in mental health, quality of life, and caregiver burden among informal caregivers during the second wave of the COVID-19 pandemic in Germany: a representative, population-based study. Gerontology 69(2):149–162. 10.1159/000523846 35390788 10.1159/000523846 PMC9148891

